# Greater loss of mitochondrial function with ageing is associated with earlier onset of sarcopenia in *C. elegans*

**DOI:** 10.18632/aging.101654

**Published:** 2018-11-19

**Authors:** Christopher J. Gaffney, Amelia Pollard, Thomas F. Barratt, Dumitru Constantin-Teodosiu, Paul L. Greenhaff, Nathaniel J. Szewczyk

**Affiliations:** 1 MRC/ARUK Centre for Musculoskeletal Ageing Research, NIHR Nottingham BRC, University of Nottingham, Nottingham, UK; 2 Lancaster University Medical School, Lancaster University, Lancaster, UK

**Keywords:** sarcopenia, ageing, muscle, *C. elegans*, mitochondria, ATP production, sarcomere

## Abstract

Sarcopenia, the age-related decline of muscle, is a significant and growing public health burden. *C. elegans*, a model organism for investigating the mechanisms of ageing, also displays sarcopenia, but the underlying mechanism(s) remain elusive. Here, we use *C. elegans* natural scaling of lifespan in response to temperature to examine the relationship between mitochondrial content, mitochondrial function, and sarcopenia. Mitochondrial content and maximal mitochondrial ATP production rates (MAPR) display an inverse relationship to lifespan, while onset of MAPR decline displays a direct relationship. Muscle mitochondrial structure, sarcomere structure, and movement decline also display a direct relationship with longevity. Notably, the decline in mitochondrial network structure occurs earlier than sarcomere decline, and correlates more strongly with loss of movement, and scales with lifespan. These results suggest that mitochondrial function is critical in the ageing process and more robustly explains the onset and progression of sarcopenia than loss of sarcomere structure.

## Introduction

Sarcopenia is the age-related loss of muscle mass, function and quality that impairs quality of life and is a strong predictor of disability. It is also a predisposing factor to other medical conditions, falls and early mortality [1,2]. Sarcopenia, therefore, represents a significant financial burden for public health providers, and in keeping with this, the Medical Research Council (MRC) have estimated that the cost of musculoskeletal decline to the UK was £5.7 billion in 2012. Moreover, worldwide population is ageing such that it is predicted that there will be over 1.5 billion people over 65 years old by 2050 [3], and sarcopenia will increase in line with this ageing population.

Sarcopenia is not exclusive to humans, and has been observed in non-human primates, dogs, rodents and even the microscopic worm, *C. elegans*. These observations, therefore, suggest that sarcopenia is an evolutionarily conserved process and whilst some evidence suggests the underlying mechanism(s) might also be conserved, it remains an open question [4–6]. There are several theories regarding the cause of sarcopenia (for a review see [7]), but we do not yet fully understand its aetiology, not least because of an absence of life-long, prospective studies. Studying the progression of sarcopenia across the lifespan poses technical challenges in animal models and humans alike. The last two decades have seen tremendous growth in ageing research employing model organisms including the nematode *C. elegans*. It has a short lifespan of 2-3 weeks at 20°C, is relatively inexpensive to work with and has emerged as a powerful genomic tool for studying ageing. Indeed, research in *C. elegans* has led to discoveries including the regulators of the insulin signaling pathway [8], the cell death machinery [9] and genes which regulate longevity [8].

Muscle architecture is highly conserved between *C. elegans* and mammals [10] and the major signaling pathways [11] and degradation systems are also present in both system [12]. Thus, *C. elegans* is a good organism in which to investigate the molecular changes to muscle with ageing. Previous studies have shown that ageing in *C. elegans* muscle is characterized by altered structure and reduced function [5,6,13,14]. This is displayed as progressive disorganization of sarcomeres and reduced cell size [6,13]. Alterations to sarcomere structure have been associated with changes to locomotive ability [6]. Alongside changes to muscle structure and function, mitochondrial defects such as increased fragmentation and reduced mitochondrial volume have also been observed in the body wall muscles of aged *C. elegans* [15].

Recently large scale studies using RNAi have been conducted to investigate how muscle health is maintained in *C. elegans* [16–19]. These studies have examined the effect of knocking down more than 850 genes (approximately 4% of the *C. elegans* genome) on sub-cellular muscle architecture. The results highlighted that in control animals sub-cellular components remained normal through early adulthood, however, after day three of adulthood, abnormal sarcomere and mitochondrial structures were observed [16]. Furthermore, mitochondrial fragmentation appeared to arise earlier in the ageing process than the alterations to sarcomere structure. These data suggest that mitochondrial abnormalities precede other changes to muscles with age. However, there has not yet been a detailed prospective study conducted to delve into the sequence of events.

This study, therefore, aimed to systematically characterize sub-cellular and whole muscle changes with age. We examined the relationship between mitochondrial structure and function and age-related muscle decline under altered natural growth conditions and lifespan. Specifically, we sought to determine the temporal associations between maximal mitochondrial ATP production rates (MAPR), mitochondrial content, mitochondrial network structure, age-related movement decline and the loss of sarcomere structure in *C. elegans*.

## RESULTS

### Loss of mitochondrial-network structure precedes the loss of sarcomere structure in ageing *C. elegans* muscle at 20°C

**Figure 1:**
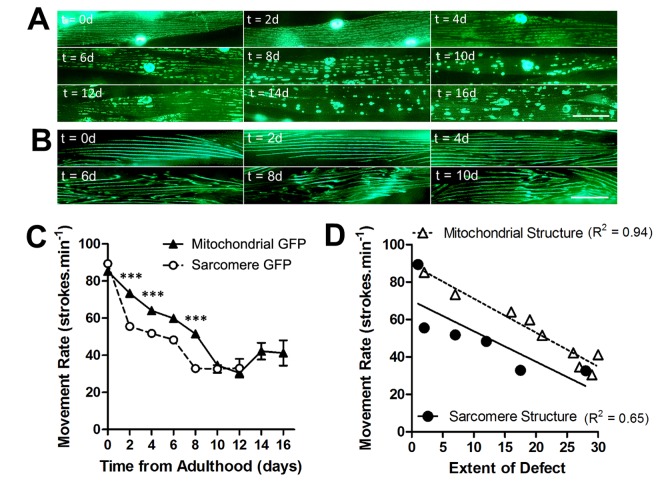
**Loss of mitochondrial structure is an early ageing event and is strongly associated with age-related loss of movement.** (**A**) Age-associated loss of mitochondrial network structure in animals grown at 20°C. Networks are retained until 4d when minor fragmentation is visible before moderate (6d) and severe fragmentation (8d) ensues. (**B**) In contrast, sarcomeres retain their structure until 6d when there is a loss of architecture. (**C)** There is a faster decline in movement rates in animals with mitochondrial GFP than with sarcomere GFP. (**D**) When correlating extent of defect in each subcellular compartment and the movement decline, the decline in movement showed greater association with loss of mitochondrial (R^2^ = 0.94) than sarcomere (R^2^ = 0.65) structure. Scale bars in A and B represent 25 μm.

We assessed mitochondrial network structure and sarcomere structure in the muscle of animals at 20°C across the lifespan, to determine the rate of age-related decline in each subcellular compartment. In animals with GFP localized to mitochondria and nuclei in muscle, mitochondria were networked from 0d - 2d of adulthood, but began to fragment from 4d onwards (Figure 1A). At 10d of adulthood and beyond, mitochondrial networks were severely fragmented in the majority of the population (Figure 1A). In animals with GFP localized to myosin, a major contractile protein in muscle, the sarcomeres appear as straight lines of GFP from 0d- 4d of adulthood (Figure 1B). It was not until 6d and beyond that animals developed minor disruption, which was further exacerbated by 8d where the majority of animals had moderate or severe defects in the sarcomeres.

### Age-related movement decline associates more with disruption of muscle mitochondrial network structure than with disruption of sarcomere structure

*C. elegans* is a widely-used model organism for investigating the mechanisms of ageing and features an age-related movement decline; one of the clearest indicators of age-associated muscle decline (Figure 1C). We sought to determine whether this overt phenotype of reduced motility, which indicates muscle decline, showed a greater association with the decline in the mitochondrial network or sarcomere structure. When the age-dependent decline in movement, determined using swim assays (Figure 1C), was correlated with disruptions in mitochondrial network structure and sarcomere structure in animals, the association with mitochondrial network structure was greater (mitochondria: R^2^ = 0.94; sarcomere structure: R^2^ = 0.65; Figure 1D).

### The age-associated decline of mitochondrial networks precedes that of sarcomere structure independent of cultivation temperature

**Figure 2:**
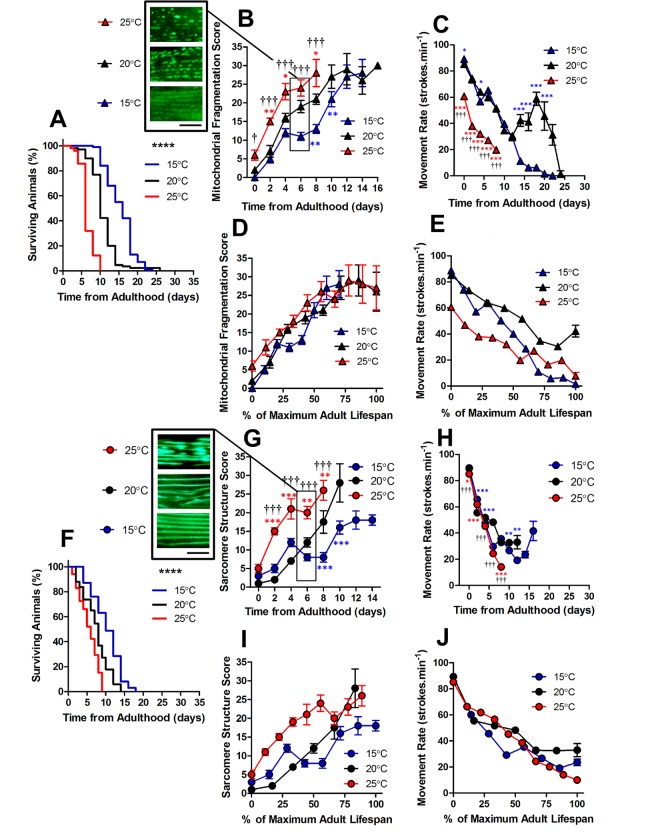
**Changes in lifespan did not change the order of progression of sub-cellular defects in *C. elegans*.** (**A**) Temperature was used to lengthen (15°C) and shorten (25°C) lifespan in comparison to control (20°C) in animals with GFP-labelled mitochondria (median lifespan of 16d at 15°C vs. 10d at 20°C vs. 6d at 25°C, P<0.0001). (**B**) Mitochondrial fragmentation is greater at 25°C and lesser at 15°C across the lifespan. (**C**) Movement across the adult lifespan in GFP-labelled mitochondria is lower in 25C than other temperatures throughout the lifespan. (**D**) When data were expressed as % maximal lifespan the temporal progression of mitochondrial fragmentation was similar between strains. (**E**) When expressed as % maximal lifespan, the movement decline between 20°C and 25°C was similar to 50% of maximal lifespan. (**F**) Temperature was used to lengthen (15°C) and shorten (25°C) lifespan in comparison to control (20°C) in animals with GFP-labelled sarcomeres (median lifespan of 10d at 15°C vs. 8d at 20°C vs. 6d at 25°C, P<0.0001). (**G**) Decline in sarcomere structure was accelerated in animals at 25°C across the lifespan and sarcomere structure was preserved in 15°C compared to 20°C from 6d onwards. (**H**) Movement rates in animals with GFP-labelled sarcomeres were lower in animals at 25°C throughout the lifespan (**I**) Expressing data as % maximal lifespan did not appear to change the relationship between defect progression at different temperatures. (**J**) Movement expressed as % maximal lifespan shows that the rate of decline scales to lifespan. All data are presented as mean ± SEM. Scale bars in (**B**) and (**G**) represent 10 μm.

*C. elegans* are poikilotherms, therefore changing cultivation temperature can change both metabolic rate (15°C = ~37 nW; 20°C = ~62 nW; 25°C = ~71 nW at young adulthood) and lifespan [20]. We used cultivation temperature as a tool to investigate the changes to mitochondrial and sarcomere structure with altered lifespan*.* Previous observations have shown that longevity mutants exhibit varying rates of muscle decline with ageing [14]. Therefore, we were interested to determine whether the observed changes to sub-cellular architecture were specific to environmental conditions or whether they are reproducible features of natural ageing. We cultivated *C. elegans* at 15°C which increased the lifespan and at 25°C which reduced the lifespan (both P <0.0001; Figure 2).

Using GFP expressed in muscle mitochondria [21], we found that the structure of mitochondrial networks were more fragmented in animals cultured at 25°C than 20°C both at young adulthood (0 d; P<0.05) and throughout the lifespan (Figure 2B and 4D-F). The observation that mitochondria are similarly networked in animals cultured at 15°C and 20°C, but are fragmented at 25°C, is consistent with 25°C lying beyond the physiological temperature of *C. elegans*. Since we saw mitochondrial changes at different cultivation temperatures, we were interested in whether this scaled with lifespan. We found that alterations to mitochondrial structure do indeed scale with maximum lifespan (Figure 2D). This is consistent with a previous study which has shown that ageing processes scale temporally to lifespan [22].

In animals with GFP labelled myosin, there was no significant difference in sarcomere structure between animals cultured at 15°C, 20°C and 25°C when at young adulthood (P>0.05). However, at 2d of adulthood there was a significant loss of sarcomere structure in the animals cultured at 25°C, but not those cultured at 20°C or 15°C (Figure 2G and 4A-C). By 6d of adulthood, sarcomere structure was significantly disrupted at 25°C compared with 20°C and 15°C, that were not significantly different from each other (P>0.05; Figure 2G and 4A-C). The accelerated decline in sarcomere structure at 25°C was associated with lower movement rates at young adulthood (Figure 2H). In contrast to mitochondrial network structure (Figure 2D), the age-related disruption of sarcomere structure did not scale with lifespan (Figure 2I).

### Protein degradation occurs after mitochondrial fragmentation with age in *C. elegans*

**Figure 3:**
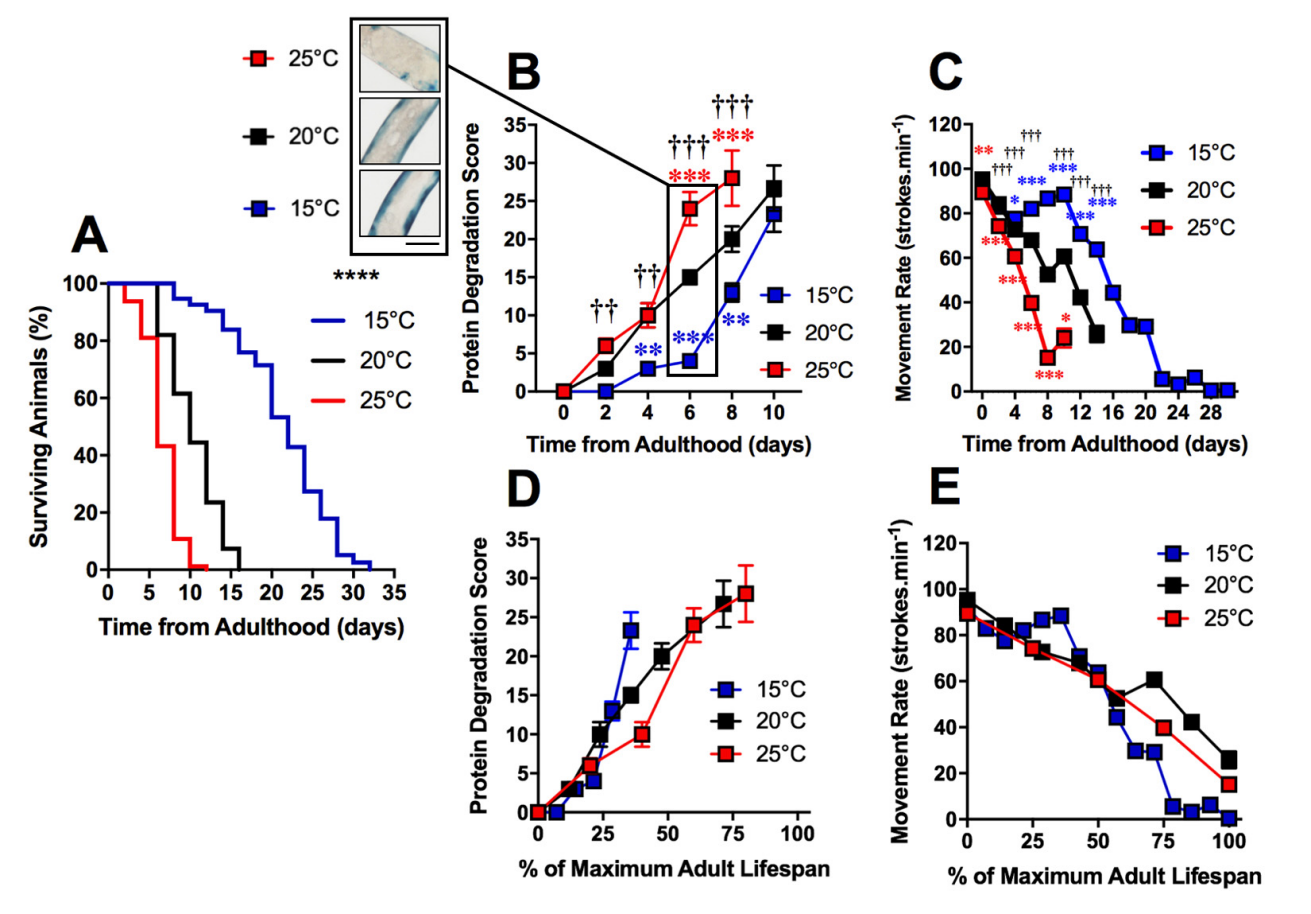
**Protein degradation is accelerated in shorter-lived and delayed in longer-lived animals.** (**A**) Temperature was used to lengthen (15°C) and shorten (25°C) lifespan in comparison to control (20°C) in animals with Lac-Z transgene (median lifespan of 22d at 15°C vs. 10d at 20°C vs. 6d at 25°C, P<0.0001). (**B**) The onset of increased protein degradation appears higher in 25°C animals and is delayed in 15°C animals. At 6d, insert shows that protein homeostasis is maintained at 15°C, somewhat maintained at 20°C and lost at 15°C. (**C**) Movement rates in animals with LacZ transgene show that movement decline is greater in 25°C animals and delayed in 15°C animals. (**D**) Rates of protein degradation expressed as % maximal lifespan shows that differences between temperatures are minimized. (**E**) Movement expressed as % maximal lifespan shows that until 50% maximal lifespan, differences between temperatures are minimized. Beyond 50%, movement rates are preserved in 20°C, but are lower throughout the remainder of the lifespan in 15°C in comparison to 25°C. Scale bar in **B** represents 80 μm.

**Figure 4:**
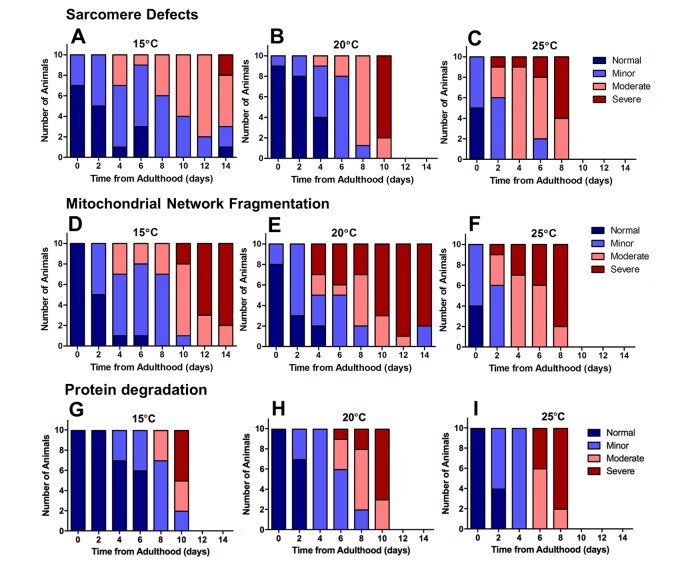
**Severe defects in mitochondrial networks were more frequent and manifested earlier in the ageing process than sarcomere defects or defects in protein homeostasis**. Data presented show individual scoring of sarcomere defects at 15°C (**A**), 20°C (**B**), and 25°C (**C**); scoring of mitochondrial network fragmentation at 15°C (**D**), 20°C (**E**), and 25°C (**F**); and protein degradation at 15°C (**G**), 20°C (**H**), and 25°C (**I**). All data represent n = 10.

The loss of proteostasis is a hallmark of the ageing process and has been characterized in *C. elegans* [23,24]. Studies have reported extensive remodeling of the proteome as well as an imbalance of proteostasis in *C. elegans* with ageing [24–26]. We investigated the timing of increased protein degradation in the ageing process to determine whether the sub-cellular changes were a result of proteostatic collapse or if these changes precede and perhaps lead to alterations to proteostasis. To study protein homeostasis, we used an established *C. elegans* model containing a myosin heavy chain *lacZ* reporter transgene [27,28]. Using this model, we were able to observe the loss of β-galactosidase, which indicates protein degradation [29,30], in the muscles of *C. elegans* cultivated at different temperatures. We observed a significant difference between protein degradation at 15°C, 20°C and 25°C when at 2d of adulthood (P<0.01 Figure 3B and 4). Animals cultivated at 15°C, with a longer lifespan, exhibited later onset of reporter protein degradation. This is consistent with previous studies which have shown longer lived species exhibit a more stable proteome [31]. Similarly to mitochondrial alterations, the onset of reporter protein degradation was also found to scale with maximum lifespan (Figure 3D) and strongly correlated with the movement decline (mitochondria: R^2^ = 0.98 at 20°C). However, the onset of reporter protein degradation occurred later in the ageing process than changes to mitochondrial structure (Figure 4D-F and G-I).

In animals with GFP labelled myosin, there was no significant difference in sarcomere structure between animals cultured at 15°C, 20°C and 25°C when at young adulthood (P>0.05). However, at 2d of adulthood there was a significant loss of sarcomere structure in the animals cultured at 25°C, but not those cultured at 20°C or 15°C (Figure 2G and 4A-C). By 6d of adulthood, sarcomere structure was significantly disrupted at 25°C compared with 20°C and 15°C, that were not significantly different from each other (P>0.05; Figure 2G and 4A-C). The accelerated decline in sarcomere structure at 25°C was associated with lower movement rates at young adulthood (Figure 2H). In contrast to mitochondrial network structure (Figure 2D), the age-related disruption of sarcomere structure did not scale with lifespan (Figure 2I).

### Longevity is associated with lower maximal mitochondrial ATP production, and lower mitochondrial content in *C. elegans*

Regardless of cultivation temperature, we observed age-related changes to mitochondrial structure and reporter protein degradation. These two factors suggest that metabolic imbalance occurs with ageing. Since we noted alterations in mitochondrial network structure earlier in the ageing process than the onset of reporter protein degradation, we investigated if there were changes to mitochondrial function. In order to do this, we first had to establish baseline mitochondrial function at different temperatures.

**Figure 5:**
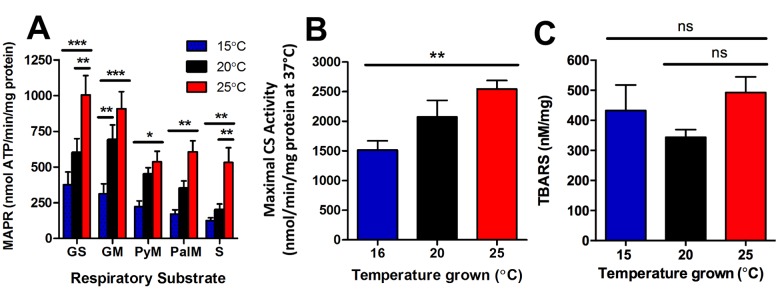
**Longevity at lower cultivation temperatures is associated with lower mitochondrial function and lower mitochondrial content.** (**A**) At the onset of young adulthood, MAPR in worms with GFP-labelled mitochondria was significantly lower at 15°C, and greater at 25°C than at 20°C. GS denotes glutamate and succinate; GM denotes glutamate and malate; PyM denotes pyruvate and malate; PalM denotes palmitoyl-L-carnitine and malate; and S denotes succinate as respiratory substrate (n = 6, P<0.05 – P<0.001). (**B**) Maximal CS activity, indicative of mitochondrial content, was lowest at 16°C and highest at 25°C (n = 8, P<0.01). (**C**) Lipid peroxidation was not significantly different between animals at different temperatures (n = 6 – 11, P >0.05). Data represent mean ± SEM, * P<0.05; ** P<0.01; *** P<0.001.

We isolated mitochondria from age-synchronised populations of worms and measured maximal mitochondrial function *ex vivo* using the reaction of ATP with firefly luciferase. We measured maximal mitochondrial ATP production rates at young adulthood, in animals grown at 15°C, 20°C, and 25°C and confirmed that the capacity to produce ATP correlated with metabolic rate [20]. In animals at young adulthood, we observed that MAPR was lowest in the 15°C animals, and highest in the 25°C animals (Figure 5A). Indeed, MAPR was significantly greater in 25°C animals than 15°C animals with all respiratory substrate combinations tested (P<0.05 – P<0.001; Figure 5A). The increased MAPR we observed could have resulted from increased mitochondrial content and/or increased intrinsic mitochondrial function per se. We therefore determined the protein content of the isolated mitochondria used to measure MAPR, and then normalized our MAPR data to this measure of mitochondrial content. This confirms an increase in intrinsic mitochondrial function, independent of mitochondrial content.

We further investigated if there were any changes in mitochondrial content of the entire worm with changes in temperature. We therefore measured maximal citrate synthase activity in young adult worms cultured at 16°C, 20°C, and 25°C to determine any differences in mitochondrial content. There was significantly lower mitochondrial content in the 16°C animals, and significantly greater mitochondrial content in the 25°C animals (P<0.01; Figure 5B). Lastly, we sought to determine if the decreased mitochondrial function and content at 15°C and increased mitochondrial function and content at 25°C, was associated with changes in reactive oxygen species that could perhaps explain subsequent differences in lifespan. Lipid-peroxidation was measured using the thiobarbituric acid reactive substances (TBARS) assay in young adult animals, at the same life-stage as the MAPR/CS assays (Figure 5A-B). There were no significant differences in lipid peroxidation between temperatures (P>0.05; Figure 5C). In summary, young adult animals cultured at 15°C have lower metabolic rates [20], lower MAPR (Figure 5A) and reduced mitochondrial content (Figure 5B), and are long-lived. In contrast, young adult animals cultured at 25°C have higher metabolic rates, increased MAPR and greater mitochondrial content, and are short lived.

### Loss of mitochondrial function precedes severe loss of mitochondrial structure with age in muscle

**Figure 6:**
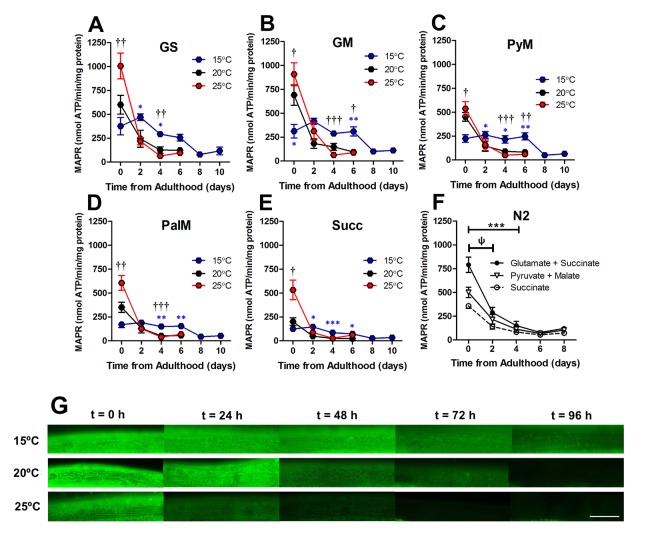
**Loss of mitochondrial function was an early event in ageing *C. elegans*.** (**A-E**) At 2 d, the absolute difference between MAPR at 15°C, 20°C, and 25°C is greatly reduced versus 0d in animals with GFP-labelled mitochondria, with a range of respiratory substrates. At 2 d, 25°C was not significantly greater than 15°C or 20°C with any respiratory substrate (P >0.05). There was a significant reduction in MAPR over time (P<0.001), primarily at 0d – 2d with 25°C (all substrates P <0.05 – P <0.001) and 20°C (all substrates P<0.05 – P<0.001) but not 15°C (all substrates P >0.05). This same loss of MAPR was observed in wild-type *C. elegans* at 20°C (GS 0 vs. 2 d, P <0.05 and all substrates P< 0.001 0 vs. 4 d). Data represent mean ± SEM, * P <0.05; **P <0.01; ***P <0.001. (**G**) Assessment of mitochondrial membrane potential using JC-10 shows that mitochondrial membrane potential is preserved at 15°C (> 96h) whereas at 25°C, mitochondrial membrane potential is lost early in the lifespan (24-48h). Scale bar represents 25 μm.

After establishing the baseline mitochondrial content and function with temperature, we investigated the alterations to mitochondrial function with age in *C. elegans.* We measured MAPR across the lifespan by sacrificing small populations of animals cultured at different temperatures at 2d intervals throughout the adult lifespan until worm number biomass prevented the measurement of MAPR. Surprisingly, at 20°C there was a marked decline in MAPR from 0 to 2d of adulthood with all mitochondrial substrate combinations after which the decline was less marked (P<0.05 – P<0.001; Figure 6A - E). A similar decline in MAPR was observed in N2, a wild-type strain with no transgenic proteins from 0 – 2d of adulthood (P<0.05; Figure 6F), suggesting that GFP used to visualize muscle mitochondrial architecture was not the cause. Similar to animals at 20°C, the magnitude of decline in intrinsic mitochondrial function at 25°C was most marked for all substrates from 0 - 2d (P <0.05 – P <0.001) and then dissipated. However, this pattern was not observed at 15°C. Whilst MAPR tended to be lower at 0d in 15°C (Figure 6A), there was not a significant decline in MAPR in these animals until 8d of adulthood (Figure 6A - E).

To confirm mitochondrial function decline with age, we used an *in vivo* dye (JC-10) to detect mitochondrial membrane potential. *C. elegans* were stained with JC-10 at 0 to 4d of adulthood at different cultivation temperatures (15°C, 20°C, and 25°C). We observed a loss of JC-10 from 0 to 4d of adulthood, which indicates that there is a loss of mitochondrial membrane potential during the ageing process (Figure 6G). This is consistent with previous data that utilized JC-10 to observe mitochondrial membrane potential at 20°C [32]. In particular, we noted that animals cultured at 15°C exhibited slower losses of the dye, with mitochondria still visibly stained at 3d of adulthood (Figure 6G). The JC-10 data supports the MAPR data, suggesting there is a delayed loss of mitochondrial function at 15d compared with animals grown at 20°C and 25°C. Furthermore, the loss of membrane potential preceded the severe defects in mitochondrial structure with age. At 15°C mitochondrial membrane potential and MAPR are maintained until 6d (Figure 6) whereas mitochondria remain networked until 10d when moderate defects become prevalent. The 20°C animals maintain mitochondrial membrane potential until 48h and MAPR appear to decline exponentially at 2d and 4d, and this is followed by loss of mitochondrial network structure at 6d. In contrast to 15°C, at 25°C mitochondrial membrane potential and MAPR are maintained only until 2d and similarly, there is a loss of mitochondrial network structure from 4d with 70% prevalence of moderate and 30% severe defects (Figure 4F). In contrast to recent observations where changes in mitochondrial structure appear to lead to changes in mitochondrial function following the disruption of the integrin-adhesome [16], the present findings suggest that with age changes in mitochondrial function lead to changes in mitochondrial structure.

## DISCUSSION

### Summary of findings

The molecular mechanisms behind the development of sarcopenia remain poorly defined. However, gene expression studies investigating human muscle with age have suggested alterations in metabolic pathways, specifically gene expression changes to the electron transport chain and insulin signaling pathway [33,34]. Here, we used the model organism *C. elegans* to prospectively investigate sub-cellular and whole muscle changes across the lifespan. Our findings show that there is increased protein degradation, reduced mitochondrial function, and disrupted mitochondrial and sarcomere structure with age in *C. elegans* muscle. We used temperature to modulate lifespan in *C. elegans* [20,35], to naturally accelerate and delay the development of sarcopenia. We see the same progression of sub-cellular changes with age at all three temperatures in *C. elegans* muscle. In particular, we found decreased mitochondrial function, followed by altered mitochondrial structure, increased protein degradation, and loss of sarcomere structure with age. Interestingly, the long-lived animals cultured at 15°C have lower MAPR and lower mitochondrial content at the onset of adulthood and display delayed development of sarcopenia in comparison to shorter-lived animals cultured at 20°C or 25°C. Moreover, these long-lived animals, with delayed development of sarcopenia, are able to preserve intrinsic mitochondrial function longer than their shorter-lived counter-partners. Indeed, the most surprising finding of the present study is that loss of mitochondrial function rather than loss of sarcomere structure appears an early event in ageing *C. elegans* muscle.

### Disrupted metabolism appears to be more of a driver of sarcopenia than cytoskeletal alterations

In *C. elegans* it has been shown that loss of proteostasis, sarcomere or mitochondrial structure are each independently associated with a movement defect [21]. In this study, the age-related decline in movement showed stronger associations with declines in mitochondrial network structure and proteostasis than declines in sarcomere structure, at all temperatures. Our data suggests that measures of mitochondrial function and proteostasis may better explain the movement decline with age rather than changes to contractile apparatus. This is supported by previous observations which have shown reduced gene expression of electron transport chain complexes in human muscle with age [33]. These findings suggest that reduced mitochondrial function is contributing to the progression of sarcopenia. Remarkably, a similar finding has recently been reported for rodent muscle as well [36]. Therefore, it may be beneficial to target improving metabolism rather than cytoskeletal components in the treatment of sarcopenia.

### Decreased mitochondrial function can lead to poor health or longevity in *C. elegans*

We have shown that reductions in mitochondrial function precede other sub-cellular defects in *C. elegans* muscle with age. Our observations are consistent with past studies in *C. elegans* where reductions in complex I and II activity [37], ATP synthesis [38], and oxygen consumption [39] have been observed with ageing at 20°C. Reductions in mitochondrial function with age have also been reported in rats [40,41], primates [42] and humans [43]. Given that mitochondrial function is required to generate the ATP required to maintain plasma membrane potential and thereby maintain cellular homeostasis, it is unsurprising that reductions in mitochondrial function lead to poor health and death in both worms and people [44]. Therefore, it is possible the declines in mitochondrial function that we observe cause the subsequent changes in muscle. It has been suggested that changes in the *C. elegans* metabolome and proteome with age may be explained by mitochondrial dysfunction [45]. Thus, it may be that the declines in mitochondrial function with age, we and others observe, are leading to poor health with age.

From a medical, cell biology, and energetics perspective, our suggestion that mitochondrial dysfunction with age may be driving declines in health is not surprising. However, studies of longevity in *C. elegans* have previously shown that longevity mutants have less mitochondria and also exhibit reduced mitochondrial function [46,47]; observations consistent with the longevity and lower mitochondrial content and function we observe in *C. elegans* cultivated at 15°C. This apparent paradox of mitochondrial dysfunction being associated with disease and longevity has previously been reviewed [44]. Briefly, it appears that mitohormesis can in part explain this paradox [48]. In *C. elegans*, the mitochondria’s fate is largely determined during development since there is a five-fold increase in mitochondrial content at the L4 stage (mtDNA copy number increases to 1.3 x 10^5^) [49]. Mild levels of stress during *C. elegans* development are thought to be protective due to several stress responses [44]. For example, RNAi knockdown during the L4 stage of electron transport chain complexes I, III, IV and V extends lifespan in *C. elegans* [50]. Conversely, severe stress during development overwhelms these protective responses and leads to mitochondrial dysfunction and subsequently death [44]. Similarly, once *C. elegans* reach adulthood, RNAi knockdown of electron transport chain complexes no longer promotes longevity but rather induces early death [50]. Thus, it seems likely that the post-adulthood declines in mitochondrial function that we observe are more likely linked to health decline with age than promoting a longevity response.

## MATERIALS AND METHODS

### Experimental protocol

Changes in movement, mitochondrial structure and function, and sarcomere structure were assessed across the lifespan in *C. elegans*. These measurements were completed at 20°C as a control, and then measurements were repeated at 15°C to increase lifespan and 25°C to decrease lifespan. This was completed to determine whether the progression of subcellular defects with age was conserved at different temporal resolutions.

### Survival analysis

Lifespan analysis was completed from young adulthood defined as t = 0 d. Worms were assessed for movement, pharyngeal pumping and response to touch, and a negative response to all assessments was defined as dead. All survival curves at t = 0d comprise of n = 100 animals. Animals were censored if accidently killed by the experimenter or if the animal desiccated itself on the side of the Petri dish.

### Movement assays

Movement rates of animals were measured prospectively using a swim test as previously described [51]. Briefly, worms were picked into a drop of M9 buffer on a microscope slide; the number of body bends in 10s were counted and then multiplied by six to give movement rates/minute. This measure was repeated five times per worm and an average rate was taken per animal.

### Assessment of β-galactosidase activity

To assess protein degradation animals were stained with X-gal. Animals were grown until day 10 of adulthood at 5°C and 20°C. Whilst animals were grown until day 8 of adulthood at 25°C. Every two days, worms were picked and stained for β-galactosidase activity as described [27].

### Microscopy and assessment of mitochondrial structure, sarcomere structure and protein degradation

All images were captured on either a Nikon H600L with a Nikon Digital Sight DS-Fi1 digital camera and proprietary software, or a Zeiss AX10 microscope with an Axiocam MRC digital camera and Axiovision LE software. Image analysis and figure preparation was conducted in GIMP and ImageJ.

Assessment of mitochondrial network structure, sarcomere structure and protein degradation was based on the method previously described [21,29]. Ten animals were imaged per time point and mitochondrial networking was determined to be normal, have minor fragmentation with small infrequent gaps in the network, moderate fragmentation with large and frequent gaps in the network or severe fragmentation where mitochondrial networks were no longer recognizable.

For the sarcomeres, animals with intact, linear sarcomeres were classed as normal, an animal with <5 muscles with disruptions in linearity was classed as minor and >5 as moderate, and animals with missing sarcomeres, bundling or large tears were classed as severe. Normal were assigned a score of ‘0’, minor ‘1’, moderate ‘2’ and severe ‘3’ which with ten animals imaged per time point gives a total score out of 30 where the larger the number the greater the disruption in that subcellular compartment. The results presented in this study have been confirmed by at least two observers from within our lab in separate experimental setups (test +24 h retest same-observer CV = 3.8% and 10.8%; and inter-observer CV = 9.0% and 14.6% for mitochondrial networks and sarcomeres, respectively).

For the protein degradation, the loss of β-galactosidase stain was scored according to severity. Animals with a score of ‘0’ were stained blue suggesting protein degradation had not occurred. Animals with a score of ‘1’ were mostly blue with a couple of clear patches suggesting protein degradation was occurring. Animals with a score of ‘2’ had a mixture of blue and clear patches. Animals with a score of ‘3’ were mostly clear suggesting protein degradation had occurred. Ten animals were stained per time point and the images were scored by two observers.

### Maximal mitochondrial ATP production assays (MAPR)

Maximal ATP production assays (MAPR) were based on the original MAPR method for human skeletal muscle [52] with modifications described previously for *C. elegans* [16]. MAPR was determined in a synchronised population of 150 - 250 animals per biological sample, and MAPR was measured until the number of remaining animals fell below ~ n = 100. At this point and beyond, MAPR could not be measured because a large enough biomass of isolated mitochondria could not be obtained to determine changes in luminescence within the linear range. Briefly, worms were washed using M9, were then homogenised in buffer (100 mM KCl, 50 mM KH_2_PO_4_, 50 mM Tris, 5 mM MgCl_2_, 1 mM EDTA, and 1.8 mM ATP at pH 7.2) for three minutes, and then mitochondria were isolated using differential centrifugation. Isolated mitochondria were washed and then resuspended in resuspension buffer (human serum albumin, 0.5 mg/ml, 240 mM sucrose, 15 mM KH_2_PO_4_, 2 mM Mg(CH_3_COO)_2_ × 4 H_2_O, and 0.5 mM EDTA at pH 7.2). Mitochondrial ATP production was determined luminometrically as previously described [16], where luciferase reagent, ADP, respiratory substrate, and mitochondria are added to wells in series, and ATP production was determined by change in luminescence in comparison to that of a known standard. Data were normalized to mitochondrial protein content determined using the Bradford method [53].

### Strains and culture

Strains of *Caenorhabditis elegans* were handled, maintained and roughly age synchronised, where stated, as previously described [27]. Strains for all experiments were cultured for a minimum of 2 generations at the experimental temperature before completing age synchronisation. Worm strains used for MAPR measurements were N2 and CB5600. CB5600 is the strain where mitochondrial structure was assessed *in vivo* and using this strain allows structure and function to be assessed in a genetically homozygous population. The worm strain used for the mitochondrial imaging experiments was CB5600: *ccls4251* (*myo-3::Ngfp-lacZ, myo-3::Mtgfp*) I; *him-8*(*e1489*) IV. This strain has GFP localized to both mitochondria and nuclei in the body wall muscles. The worm strain used for the sarcomere imaging experiments was PJ727: *jls01* (*myo-3::GFP, rol-6*(*su1006*) ?; *ccIs55* (*unc-54::lacZ*) V. This strain has GFP localized to myosin within the body wall muscles that therefore allowed visualization of the sarcomeres. The worm strain used for assaying protein degradation was PD55: *ccIs55* (*unc-54::lacZ*) V. This strain has β-galactosidase localized to the cytosol within the body wall muscle that can be used to report on increased protein degradation. Details on imaging using these strains have been described previously [21].

### Changing temperature to manipulate lifespan in *C. elegans*

*C. elegans* is a poikilotherm and this means that its internal temperature is governed by the temperature of the environment [35,54]. Changing temperature in *C. elegans* is a common method used to manipulate lifespan. By reducing temperature to 15°C, lifespan is increased, and by increasing the temperature to 25°C, the lifespan is shortened [15,35,55]. Recent research has shown a temperature dependency of mitochondrial networking in *C. elegans* [15]. This is therefore an appropriate experimental manipulation to investigate changes in sub-cellular compartments in *C. elegans* at different rates of living.

### Determination of maximal citrate synthase activity

Maximal citrate synthase activity was determined as previously described [56,57].

### Assessment of ROS production

ROS production was assessed through measuring thiobarbituric acid reactive substances (TBARS). Samples were homogenised in 50 mM Tris HCl, 1mM EDTA, 1 mM EGTA, 1% IGEPAL (pH 7.5) and 0.1% mercaptoethanol, added immediately before homogenisation. Standards were generated using 1, 1, 3, 3, tetramethoxypropane (TMP). One hundred µl of sample was added to 750 µl of 20% acetic acid (pH 3.5), 100 µl of 8.1% SDS and 1050 µl of 0.57% thiobarbituric acid (TBA). Samples were vortexed and were then heated at 100°C for 1 h. After boiling, samples were cooled on ice for 10 min, and then 2 ml n-butanol-pyridine (15:1 v/v) was added. Samples were then centrifuged at 1,500 g for 10 min. The butanol phase was removed and was measured in a fluorescence spectrophotometer at 553/515 nm (Ex/Em).

### Data expressed as a percent of maximum lifespan

This study sought to determine the order which muscle defects manifest during the ageing process. Since the strains used to examine the sarcomeres and mitochondria have different lifespans it was useful to express data as a percentage of maximum lifespan. This allowed the progression of the defect to be recorded in relative terms to the strain used for that investigation. The point where ≥ 90% animals had died was used as maximum lifespan when data were corrected this way. To express data as a percent maximum lifespan, the time point where 90% of animals had died was used to normalize data to lifespan. This was to remove survivor effects from the data and obtain maximum lifespan representative for the initial 90% of animals.

### *In vivo* assessment of mitochondrial membrane potential using JC-10

To assess mitochondrial membrane potential the *in-vivo* dye JC-10 (Enzo Life Sciences 52305) was used. Wild-type worms were cultured at 15°C, 20°C and 25°C and stained with JC-10 at 0, 24, 48 and 72 hours of adulthood. The worms were picked into 83 μM of JC-10 in freeze dried OP50 solution (LabTIE) for 4 hours before imaging. Representative images are shown for each time point.

### Statistical analyses

All data for the MAPR and CS are presented as means ± SEM from at least four replicates unless otherwise stated. Statistical differences were assessed using either one-way ANOVA with Newman-Keuls corrections or two-way ANOVA with Bonferroni corrections. Non-parametric data was log transformed and analysed using two-way ANOVA. Survival curves represent a single experiment using n = 100 animals. Survival curves were assessed for significance using Kaplan-Meier survival analysis. Significance was determined as P<0.05 and all statistics were completed using GraphPad Prism (USA).
